# Genome-scale cold stress response regulatory networks in ten *Arabidopsis thaliana* ecotypes

**DOI:** 10.1186/1471-2164-14-722

**Published:** 2013-10-22

**Authors:** Pankaj Barah, Naresh Doni Jayavelu, Simon Rasmussen, Henrik Bjørn Nielsen, John Mundy, Atle M Bones

**Affiliations:** 1 Department of Biology, Norwegian University of Science and Technology, Trondheim N-7491, Norway; 2 Department of Chemical Engineering, Norwegian University of Science and Technology, Trondheim N-7491, Norway; 3 Center for Biological Sequence Analysis, Department of Systems Biology, Technical University of Denmark, Lyngby DK 2800, Denmark; 4 Department of Biology, University of Copenhagen, Copenhagen DK-2200, Denmark

**Keywords:** *Arabidopsis thaliana*, Ecotypes, Cold stress, Natural variation, Adaptation, Gene expression, Regulatory networks, *Arabidopsis thaliana* 1001 genome, Systems biology, Network component analysis

## Abstract

**Background:**

Low temperature leads to major crop losses every year. Although several studies have been conducted focusing on diversity of cold tolerance level in multiple phenotypically divergent *Arabidopsis thaliana (A. thaliana)* ecotypes, genome-scale molecular understanding is still lacking.

**Results:**

In this study, we report genome-scale transcript response diversity of 10 *A. thaliana* ecotypes originating from different geographical locations to non-freezing cold stress (10°C). To analyze the transcriptional response diversity, we initially compared transcriptome changes in all 10 ecotypes using Arabidopsis NimbleGen ATH6 microarrays. In total 6061 transcripts were significantly cold regulated (p < 0.01) in 10 ecotypes, including 498 transcription factors and 315 transposable elements. The majority of the transcripts (75%) showed ecotype specific expression pattern. By using sequence data available from *Arabidopsis thaliana 1001* genome project, we further investigated sequence polymorphisms in the core cold stress regulon genes. Significant numbers of non-synonymous amino acid changes were observed in the coding region of the CBF regulon genes. Considering the limited knowledge about regulatory interactions between transcription factors and their target genes in the model plant *A. thaliana*, we have adopted a powerful systems genetics approach- Network Component Analysis (NCA) to construct an *in-silico* transcriptional regulatory network model during response to cold stress. The resulting regulatory network contained 1,275 nodes and 7,720 connections, with 178 transcription factors and 1,331 target genes.

**Conclusions:**

*A. thaliana* ecotypes exhibit considerable variation in transcriptome level responses to non-freezing cold stress treatment. Ecotype specific transcripts and related gene ontology (GO) categories were identified to delineate natural variation of cold stress regulated differential gene expression in the model plant *A. thaliana*. The predicted regulatory network model was able to identify new ecotype specific transcription factors and their regulatory interactions, which might be crucial for their local geographic adaptation to cold temperature. Additionally, since the approach presented here is general, it could be adapted to study networks regulating biological process in any biological systems.

## Background

Being sessile organisms, plants have evolved strategies to survive in unfavourable environmental conditions. Intraspecific variation in response to environmental stresses is clearly visible among plant species [1-4]. Understanding the molecular basis of such local adaption to complex environmental conditions has proven to be very useful in selecting better traits or target genes for modern plant sciences [5]. Cold stress is a naturally occurring hazard to world crop production. Cold stress contributes to poor germination, stunted seedlings, chlorosis, reduced leaf expansion and wilting, and may also lead to death of tissue (necrosis) [6]. Exposure to cold stress also slows down the reproductive development of plants. Plants perceive cold by the receptor at the cell membrane and a signal is initiated to activate the cold-responsive genes and transcription factors for mediating stress tolerance [7,8]. The CBF cold response pathway has a major role in cold response, tolerance and acclimation; however, considerable differences in the sets of cold regulated genes were observed [9]. *CBF* genes are induced after just few minutes of cold exposure. They encode a small family of transcription factors known as CBF1, CBF2, and CBF3 (also known as DREB1B, DREB1C and DREB1A). Cold induction of *CBF* genes regulates a set of about 100 downstream genes. Among them, the immediate target genes of CBF1-3 include *CRT* (*C-repeat*)/*DRE* (*dehydration responsive element*) elements in their promoter regions. CBF1-3 proteins bind to this DNA regulatory sequence. The dehydration-responsive element (DRE) is also known as low temperature response element (LTRE), which contributes to cold responsiveness [10]. Interestingly, induction of the CBF regulon enhances both cold and drought tolerance [11]. Earlier transcriptome profiling studies have shown that multiple regulatory pathways are activated in *A. thaliana* during cold exposure in addition to the CBF cold-response pathway [12].

Natural variation for cold response and tolerance is an important element of adaptation and geographic distribution of plant species. There is clear association between plasticity of gene expression and adaptability of an organism [13]. There have been several studies focusing on diversity of cold tolerance level in multiple phenotypically divergent *A. thaliana* ecotypes [14-16]. McKhann *et al*. reported that *CBF* and *COR* (Cold Regulated) genes respond differently to cold stress in eight accessions, though they could not find clear correlation between gene expression, sequence polymorphism and cold tolerance [17]. However, the molecular basis of the natural variation during cold stress response in plants at genome scale is not fully understood yet.

Transcriptional profiling has become a major tool to identify genes exhibiting transcriptional regulation in plants as an effect of changing environmental conditions taking *Arabidopsis* as a model system [18]. Variation in experimental conditions and protocols makes it difficult to extract and compare information from data sets produced by individual laboratories [19]. To overcome such problems, we subjected 10 ecotypes of *A. thaliana* to 5 individual stress treatments and 6 combinations of these stress treatments under the same experimental set up and profiling protocols [20]. We have considered all the cold experiments conducted on 10 ecotypes from this already published dataset (GEO accession GSE41935), to explore genome-scale transcriptomic response signatures of *A. thaliana* during cold stress treatment. By utilising data available from recently published *A. thaliana* 1001 genome project, we further analysed sequence polymorphisms in the CBF regulon genes [21].

It is likely that differential expressions or variation in mRNA stability caused by coding sequence polymorphisms significantly contribute to natural variation in *A. thaliana*[22]. Information about differentially regulated genes during different stress conditions is often available as an outcome of microarray experiments. However, in many cases, little is known about the regulation and interaction of these genes [23]. Being highly dynamic in nature, any biological system continuously changes responding to environmental and genetic perturbations. Differential dynamic network mapping of facilitates the exploration of previously unknown interactions [24]. While the *A. thaliana* genome has ~1922 TFs [25], experimentally confirmed regulatory relations are available for less than 100 TFs only (as per information extracted from the AGRIS database, version updated on September 10th, 2012) [26]. Tirosh *et al.* explained how regulatory relationships can also be deduced from patterns of evolutionary divergence in molecular properties such as gene expression [27]. To compensate for the lack of information on transcription factor activity at the genome scale, several computational algorithms have been developed to identify regulatory modules and their condition-specific regulators from gene expression data [28-30]. Network Component Analysis (NCA) is such an approach, which has been successfully implemented in several species including *A. thaliana*, to determine both activities and regulatory influences for a set of transcription factors on target genes in various perspectives [31-33]. By taking the advantage of the NCA method, we predict ecotype specific regulatory relationships, which provide new information towards understanding the natural variation in cold response pattern among different ecotypes of the model plant *A. thaliana.*

## Methods

### Microarray data

We have considered all the cold stress microarray experiments conducted on 10 ecotypes during the ERA-PG Multi-stress project [20], to explore genome-scale transcriptomic response signatures of *A. thaliana* during cold stress treatment (microarray data available at GEO with the accession GSE41935). All the experiments of ERA-PG Multistress project were set up in environmentally controlled rooms at the plant growth facilities at RISØ DTU National Laboratory for Sustainable Energy (Roskilde, Denmark). A pilot study using wild type plants Col and L*er* was set up to find the appropriate conditions at sub-lethal doses [20]. These initial observations indicated that an optimal time before the onset of a phenotypic response (e.g.: wilting, dehydration) while avoiding tissue damage was 3 hours. Ten *A. thaliana* wild ecotypes (Table 1) were grown in soil under long day photoperiod and 24°C in a greenhouse setting for one generation to amplify homogeneous seed for all different genotypes. The obtained seeds were sown into trays and grown in a Conviron growth chamber (Winnipeg, Manitoba, Canada) under a 12 hr/12 hr photoperiod, 24°C and standard *A. thaliana* growth conditions. Three week-old plants were then placed for three hours into the environmentally controlled growth rooms that were preset to cold stress conditions (10°C). Triplicated (biological) trays with the wild type controls were subject to the cold treatment. After the stress treatments, leaves tissue were collected and promptly frozen in liquid nitrogen for subsequent microarray experiments.

**Table 1:** Summary of the ecotypes and their gene expression pattern during cold treatment

**Ecotypes**			**Differentially regulated transcripts**					
**Ecotype name**	*Geographic origin	**Latitude (°North)**	**Total**	**Total up**	**Total down**	**Unique total**	**Unique up**	**Unique down**
Cvi	Cape Verdia Islands	16	2004	603	1401	1230	276	954
Kas-1	Kashmir, India	34	1097	487	610	442	153	289
Kyo-2	Kyoto city, western part of Hoshu Island, Japan	35.5	877	458	419	305	104	201
Sha	Shakdara, Tadjikistan	39	620	215	405	268	70	198
Col-0	Columbia, United States	38.5	185	120	65	89	48	41
Kond	Kondara, Tadjikistan	38.8	814	428	386	384	167	217
C24	Coimbra, Portugal	40	1427	931	496	758	460	208
L*er*	Landsberg, Poland	48	619	195	424	348	91	257
An-1	Antwerpern, Belgium	51.5	632	308	324	188	69	119
Eri	Erigsboda, Sweden	56	967	804	163	541	427	114

*Geographic origins of the ecotypes were collected from the donor , Arabidopsis 1001 Genome project database [75] and Swindell et al. [13].

This table represents geographic distribution of the 10 *A. thaliana* ecotypes and number of cold regulated genes in each of the ecotypes (p ≤ 0.01). Up and down regulation was calculated based on fold change ratios compared to respective untreated controls in individual ecotypes. Ecotype Col-0, which is a cold tolerant ecotype have very less number of cold regulated transcripts compare to others. On the other hand Cvi, the southernmost accession from a warm climate, exhibits more number of cold responsive transcripts. (Unique = Unique to the respective ecotype).

### Statistical analysis of the data

Resulting data from the microarray experiments was pre-processed using the RMA [34] implementation in the oligo package [35] in R programming platform [36]. Gene annotation was acquired from TAIR10 [37] using biomaRt data mining tool [38]. Differentially expressed genes between control and treated plants were identified using t-test (p < 0.01). Genotype specific responses to stress were identified by the interaction effect from a two-way ANOVA [39,40] of the genotype and treatment effect (p < 0.01). The union of stress responsive genes was further used for network-based analysis. Heat maps were plotted using TM4 microarray software suite [41].

### Gene set enrichment analysis (GSEA)

The Biological Networks Gene Ontology (BiNGO) tool [42], an open-source Java tool was used to determine which Gene Ontology (GO) terms [43] that were significantly overrepresented in our differentially regulated gene lists (p-values were Bonferroni corrected).

### Sequence analysis

Sequences for CBFs, and COR genes were downloaded from *A. thaliana* 1001 Genome project (http://signal.salk.edu/). Initially sequences from all available ecotypes in the 1001 genome database (706) were downloaded, but incomplete sequences were discarded before the analysis. Apart from the coding regions, we have considered 1,000 bp upstream sequences for alignment. All positions containing gaps and missing data were eliminated. Multiple sequence analysis performed using Clustal w [44]. Tajima’s D [45] statistical test to identify sequences which do not fit the neutral theory model at equilibrium between mutation and genetic drift were performed using MEGA5 suit [46].

### Network component analysis and network reconstruction

Network component analysis is a computational method for reconstructing the hidden regulatory signals (TFAs) from gene expression data with known connectivity information in terms of matrix decomposition [31,47]. NCA model assumes the log-linear relationship between target genes expression profiles and TFAs:

(1) $$\frac{E_{i}\left(t\right)}{E_{i}\left(0\right)}=\prod_{j=1}^{L}{\left(\frac{\mathrm{TF}A_{j}\left(t\right)}{\mathrm{TF}A_{j}\left(0\right)}\right)}^{CS_{\mathrm{ij}}}$$

Where *E*_*i*_(*t*) and *E*_i_(0) are the expression values of gene i at different measurement conditions and reference point 0, and similarly *TFA*_*j*_(t) and *TFA*_*j*_(0) are the activities of *TF*_*j*_, and *CS*_*ij*_ represents the control strength of TF *j* on gene *i*. Taking logarithms, the equation (1) becomes:

(2) $$\text{log}\left[\mathrm{Er}\right]=\left[\mathrm{CS}\right]\text{log}\left[\text{TFAr}\right]$$

Where the matrix *Er* represents the expression values of genes at different measurement conditions, matrix *CS* is the control strength of each TF on each TG and *TFAr* represents the TFAs of all the TFs. The dimensions of [*Er*] is N × M (N is the number of genes and M is the number of measurement conditions), [*CS*] is N × L (L is the number of TFs), and for [*TFAr*] is L × M. We can further simplify the above equation (2) as:

(3) $$\left[E\right]=\left[C\right]\left[T\right]$$

Here expression matrix [E] corresponds to [Er] in equation (2), connectivity strength matrix [C] is equivalent to [CS] and transcription factor activity matrix [T] corresponds to log[TFAr] in equation (2). Based on above formulation, the decomposition of [E] into [C] and [T] can be achieved by minimizing the following objective function:

(4) $$\text{min}||\left(\left[E\right]-\left[C\right]\left[T\right]\right)||$$

s.t. C ε Z0

Here Z0 is the initial connectivity pattern. The estimation of [C] and [T] is performed by using a two-step least-squares algorithm and normalized through a nonsingular matrix [S] according to,

(5) $$\left[E\right]=\left[C\right]\left[T\right]=\left[C\right]\left[S\right]\left[S^{-1}\right]\left[T\right]$$

In order to guarantee uniqueness of the solution for the equation (4) up to a scaling factor, there are certain criteria to be satisfied termed as NCA criteria: (a) The connectivity matrix [C] must have full-column rank. (b) When a node in the regulatory layer is removed along with all of the output nodes connected to it, the resulting network must be characterized by a connectivity matrix that still has full-column rank. (c) T matrix must have full row rank.

The algorithm for NCA analysis is implemented in MATLAB by Liao and his colleagues and it is available online for download (http://www.seas.ucla.edu/~liaoj/downloads.html). With NCA as reconstruction method, we predicted significant TFs and connectivity strength on target genes and TFAs of TFs.

## Results and discussion

### Different transcriptome signatures of 10 *Arabidopsis* ecotypes responding to cold stress

To cover a wide array of phenotypic variation, 10 natural accessions of *A. thaliana* representing habitats from 16° to 56.5° northern latitude were selected during the ERA-PG Multi-stress project. These accessions or ecotypes were- Cvi (Cape Verde Islands), Kas-1 (Kashmir, India), Kyo-2 (Kyoto, Japan), Sha (Shakdara, Tadjikistan), Col-0 (Columbia, USA), Kond (Kondara, Tadjikistan), C24 (Coimbra, Portugal), L*er* (Landsberg, Poland), An-1 (Antwerpe, Belgium), Eri (Erigsboda, Sweden) (details in Table 1). We have chosen cut-off p ≤ 0.01 to define a gene as differentially regulated. Using the results from these ten ecotypes, we were able to examine the transcriptional differences that occurred during early hours of cold treatment. The results (Table 1) indicated that *A. thaliana* ecotypes have visibly different transcriptome signatures in response to cold stress. Variable numbers of transcripts were up or down regulated by cold stress. Considering the two extreme ecotypes, Col-0 being known as cold tolerant ecotype had significantly less number of differentially regulated transcripts, while Cvi being the southernmost ecotype (among the 10 used in our experiments) had the highest number of differentially regulated transcripts. Ecotype Cvi (Cape Verde Islands) was associated with a climate temperature comparatively higher than that of the ecotype Col-0, and fact was well reflected in their transcriptional responses to cold treatment. Similar results were also reported by earlier [13].

**Figure 1:**
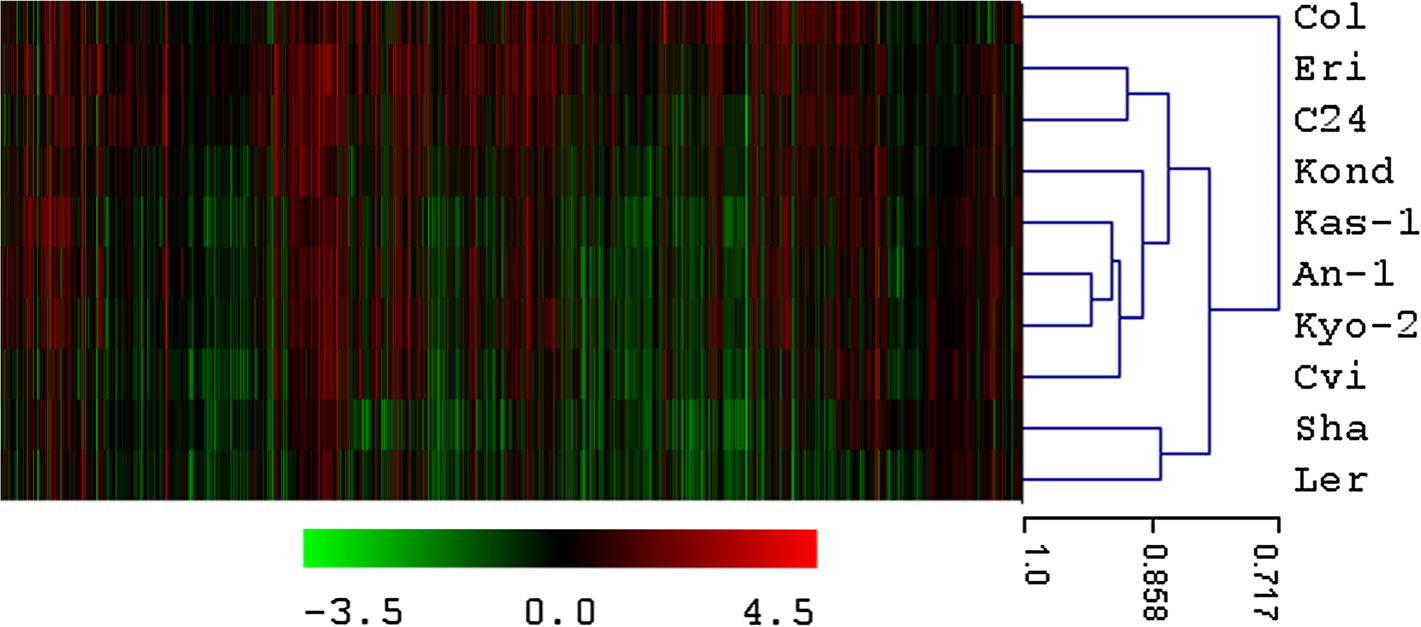
**Heat map visualization of the cold transcriptome of the ten ecotypes.** The heat map visualizes hierarchical clustering (with Pearson’s correlation coefficient using average linkage method) of top 1000 cold regulated transcripts based on gene expression fold change ratios compared to their respective controls from 10 different ecotypes. Genes are shown as columns and ecotypes are shown as rows. As a global observation, this heat map indicates differential regulation signatures in response to non-freezing cold treatment in different *A. thaliana* ecotypes. Cold tolerant ecotype Col-0 ecotype is distinctly separated out from others.

A unified list of 6061 cold regulated transcripts (p < 0.01) was generated from all the 10 ecotypes (Additional file 1). The total number of differentially regulated TFs in this list was 498. Interestingly, 4553 (75%) transcripts were differentially regulated only in one of the ten ecotypes. The significant list of differentially regulated transcripts includes most of the known cold regulated genes. Figure 1 displays fold change values (treatment vs. control) calculated from normalized expression index for top 1000 significant genes from all the 10 ecotypes. Global observation of the heat map indicates differentially regulated transcriptome signatures in response to non-freezing cold treatment in ten different *A. thaliana* ecotypes. Hierarchical clustering (HCL) was performed with Pearson correlation using average linkage method and 10,000 bootstrapping for the top 1000 cold regulated transcripts based on fold change ratios with respects to their respective controls. Ecotype Col-0 is distinctly separated out from others. Southern ecotypes Cvi, Sha and Kyo2 were grouped closely. Zhen et al. [16] has reported earlier a positive correlation between freezing tolerance and latitude of origin, based on physiological data collected from 71 *A. thaliana* ecotypes. Hannah *et al*. [48] used 9 accessions of *A. thaliana* to show that cold tolerance of natural accessions correlates with habitat winter temperatures. Clustering of the gene expression pattern in response to non-freezing cold stress exposure in ten ecotypes during our analysis doesn’t reflect a clear latitudinal trend.

### Ecotype specific cold regulated transcript lists contain many transcription factors (TFs) and transposable elements (TEs)

In contrast to the relatively small number of transcripts with altered expression shared by all the ten ecotypes, majority of the transcripts (75%) showed ecotype specific expression pattern (Additional file 2). Each of the ecotypes had unique sets of differentially regulated transcripts in response to cold stress. From the list of differentially regulated transcripts, it was found that 498 encoded for Arabidopsis TFs and 320 TFs (~ 64% of all the differentially regulated TFs) were differentially regulated in single ecotypes. The ecotype specific differentially cold regulated TFs are listed in Table 2. The list of differentially regulated transcripts includes many well-known cold regulators like *CBF*s, *DREB1A, DREB1B, DREB2B, RAV1, ERF2,* and *ERF5*. We have surveyed existing available transcription factor - target gene (TF-TG) regulatory interactions available in public databases and literature. There were 59 TFs reported as associated to cold responses in GO (Gene Ontology) database and TAIR (The Arabidopsis Information Resources). Unfortunately, none of them were included in the AtRegNet server which contained experimentally validated regulatory interactions for only 69 out of nearly 1922 known Arabidopsis TFs.

**Table 2:** Cold regulated transcription factors

**Ecotype**	**Unique TFs (Up regulated)**	**Unique TFs (Down regulated)**
**Cvi**	ANAC014, ANAC042, ANAC058, AtHB24, AtHB32, AtMYB103, CUC1, HEC1, HSFB2A, LBD27, LBD35, LD, PIL6, SRL2, At3g20880, At4g00150, At1g09060, At3g11450, At5g45270	AIL6, ANAC041, ANAC074, ANAC103, ARR2, ASML2, AtHB23, AtMYB11, AtMYB17, AtMYB86, AtNAC3, AtY13, bt5, EMB3008, ETC1, GNC, HAt22, HDG12, IAA18, LBD23, MAF4, MYB113, MYB3, MYB33, MYB92, MYC6.2, NTL9, PAN, PCF1, PUX2, SDG40, SGR1, SPL5, SUVR4, tcp17, TCP3, TGA6, VND1, WOX13, WRKY50, At1g16640, At3g06160, At4g34400, At4g00940, At5g49300, At3g57480, At5g10970, At2g05160, At5g40880, At3g16940, t5g38140, At2g20110, At1g07520, At1g63100, At1g44810, At4g00232, At4g26170, At1g09710, At1g33420, At2g01810, At3g53370, At5g51910, At1g76870, At1g26260, At1g62975, At4g00870, At4g14410, At4g29930, At5g46830, At5g65320
**Kas.1**	AtGRF3, BPC6, HAt3, HSFA8, IAA29, SSL2, SWN, WRKY3, WRKY32, WRKY66, At3g45260, At1g67310, At2g45460	AL1, BT4, DUO1, GBF6, HSFB1, HSFB4, MNP, TED5, TIFY3B, U2AF35B, ZBF1, ZFN3, ZFP4, At5G52020, At5G06770, At5G41920, At4G22140, At5G50670, At1G03040, At3G23690
**Kyo.2**	AtIDD2, CAL1, HAt14, LCL1, PHE2, RAP2.9, SNZ, SPL3, TOC1, At4g18870, At5g51790	HDT3, PMG1, TRFL6, UNE12, WER1, At5g61190, At4g23800, At2g45800, At1g69170, At1g68920, At2g46510
**Col-0**	At3G50750, At2G27630, At5G22990, At1G48195, At4G37850	ACD6, ACS3, CYP71A28, CYP81K1, MEA, MLP28, MYB24, PSRP5, RCK, STR16, XIJ, At3g21570, At5g33260, At2g21930, At5g26930, At5g15620, At3g18840, At1g31370, At2g01031, At2g09850, At2g24930, At1g79120, At4g09070, At3g56600, At5g39150, At2g35300, At1g23570, At5g02140, At1g23060, At3g14750, At1g27300, At3g16840, At3g03920, At2g07671, At1g53060, At5g66230, At5g58570, At5g26690, At1g27330, At1g18720, At5g18850
**Kond**	AGL79, ANAC077, AtGRF6, LBD14, SCL11, TIFY9 (JAZ10), WRKY10, At3g06410, At3g51950,	ADOF2, ANAC097, AtHB27, BME3-ZF, bZIP61, GIF2, HAP3A, HAP5B,ING1, RAP2.11, TGA1, At3g51080, At2g40970, At5g47390
**Sha**	AGL58, ANAC009, SET1, STY2, At4g33880	AGL24, AGL43, CRF1, ETT, IAA7, LBD38, PHB-1D, PTAC1, SRS8, ZFHD2, At5g02460, At5g41030
**C24**	ADOF1, ANAC045, ANAC061, ANAC069, AtIDD16, AtIDD5, CDF1, CDF2 , COL2 , DREB2A, HSF A4A, IAA1, LBD32, MP, MYB51, MYB77, RVE2, SPL1R2, SSRP1,SUVH3, WRKY26, WRKY33, WRKY40, WRKY55, At5g51190, At2g17410, At4g17570, At1g26610, At4g15420, At5g26610, At5g12440, At3g52250, At5g06110, At1g20640, At1g64530, At2g18090, At2g37520, At1g01260 , At5g57150	ANAC065, AtHB16, AtMYB63, BPC5,HSFB2B, LBD22, RGA2, SCL3, ZFHD3, At1g49475, At1g68520, At4g24060, At3g24050, At4g14540, At2g44730, At2g21235
**Ler**	ddf2, HSFC1	AGL26, ARF21, DAR7, emb2746, LBD1, MYB105, NAI1, ZFP6, At4g31680, At5g12850, At1g75530, At5g41765, At2g17150
**An.1**	AGL56, RD26, SOC1, SUVH1, tify5a, At1g79700, At4g15250, At2g42150,	AtGRF6, LBD14 , At3g51950
**Eri**	ALC, ANAC011, ANAC019, ANAC044, ANAC046, AtAF2, AtNAC3, AZF2, BPC4, COL9, DAG2, ERF5, ERF8, IAA17, IAA5, MYB59, MYBR1, RAP2.10, SHN3, SHY1, TIFY10B, WRKY22, WRKY27, WRKY28, ZFHD4, At4g01580, At2g40340, At2g40350, At4g32800, At2g45050, At3g49930, At3g60580, At3g08505, At3g14020, At1g25550, At3g12730, At5g01200, At5g05790, At3g21210, At3g53680, At2g18850, At3g21330, At3g23210 , At1g19490	AtbZIP,BZR2, DAG2 , WOX12, ZFP8, At3g23140, At1g19040

List of differentially cold regulated (up and down) TFs, unique for each of the 10 *A. thaliana* ecotypes (significance threshold p ≤ 0.01).

Nimblgen12-plex Arabidopsis microarray chip included 3822 transposable element (TE) probes. We have observed 315 TEs (~10% of the total TE probes printed on the chip) in the ecotype specific differentially regulated transcript list. The distribution of the differentially regulated TEs in ten ecotypes were as follows – Col-0 (21), L*er* (81), Cvi (71), Eri (31), Kas2 (16), Kond (39), Kyo2 (23), C24 (15), Sha (22) and An1 (8). Somatic events, in particular, the activity of transposable elements (TEs) do play an important role in plant genome evolution [49]. Lee *et al.*[50] reported that cold-regulated gene expression was not only controlled transcriptionally, but might also be regulated at the posttranscriptional and chromatin level [50]. A change in the epigenetic state of TEs by cold stress might contribute to regulatory activities for adjacent genes [51]. Recently Wang et al. has demonstrated that both TE sequence polymorphisms and presence of linked TEs are positively correlated with intraspecific variation in gene expression [52]. Some of the differentially regulated TEs in our cold experiments might potentially be interesting targets to explore diversity of cold stress responses among different *A. thaliana* ecotypes. Further targeted experiments in this direction can explore the molecular level details of any potential role of these TEs on genomic adaptation of the ecotypes to their local environment.

### Gene set enrichment analysis indicates activation of common and unique processes in different ecotypes

To investigate functionally relevant changes, gene ontology based overrepresentation analysis was performed using BinGO software considering the up-regulated gene lists from each of the ten ecotypes (Additional file 3). From this analysis we have created a GO attribute table by uniting all the statistically significant overrepresented GO categories from each of the ten ecotypes (Additional file 4). Genes showing significant variation in mRNA expression level in *A. thaliana* during different stress conditions mainly belong to categories like signal transduction, transcription and stress response [53]. This reflects the potential variations in the regulatory mechanisms of these genes among different ecotypes. Apart from common cold stress responsive categories like- response to cold stress, response to low temperature, cold acclimation etc., we observed few other biological processes to be differentially up-regulated in various ecotypes (Table 3). Some of the interesting and top GO categories were as follows.

**Table 3:** GO terms attribute matrix from the significantly regulated gene-list for each ecotype, generated using BiNGO software

**GO-terms**	**An.1**	**Col −0**	**Cvi**	**Eri**	**Kas.1**	**Kond**	**Kyo.2**	**Ler**	**C24**	**Sha**
Response to abiotic stimulus	✓	✓	✓	✓	✓	✓	✓	✓	✓	✓
Response to chemical stimulus	✓	✓	✓	✓	✓	✓	✓	✓	✓	✓
Response to cold	✓	✓	✓	✓	✓	✓	✓	✓	✓	✓
Response to organic substance	✓	✓	✓	✓	✓	✓	✓	✓	✓	✓
Response to endogenous stimulus	✓	✓	✓	✓	✓	✓	✓	✓	✓	✓
Response to hormone stimulus	✓	✓	✓	✓	✓	.	✓	✓	✓	✓
Circadian rhythm	✓	✓	✓	✓	✓	.	✓	✓	✓	✓
Response to light stimulus	✓	.	✓	✓	✓	.	✓	✓	✓	✓
Response to water	✓	✓	.	✓	✓	.	✓	✓	✓	✓
Response to jasmonic acid stimulus	✓	.	✓	✓	.	✓	✓	✓	✓	✓
Response to water deprivation	✓	✓	.	✓	✓	.	✓	✓	✓	✓
Response to red or far red light	✓	.	✓	✓	✓	.	✓	✓	✓	✓
Cold acclimation	✓	.	.	✓	✓	✓	✓	✓	✓	✓
Rresponse to other organism	✓	.	✓	.	✓	✓	✓	✓	✓	✓
Response to blue light	✓	.	✓	✓	✓	.	✓	✓	✓	✓
Response to abscisic acid stimulus	✓	.	✓	.	✓	✓	✓	.	✓	✓
Response to far red light	✓	.	✓	✓	✓	.	✓	✓	✓	.
Multi-organism process	✓	.	✓	.	✓	✓	✓	.	✓	✓
Response to red light	✓	.	✓	✓	✓	.	✓	✓	✓	.
Response to fungus	✓	.	✓	.	✓	.	✓	✓	✓	✓
Response to carbohydrate stimulus	✓	.	.	✓	✓	✓	✓	.	✓	✓
Regulation of transcription	✓	.	.	✓	✓	.	✓	.	✓	✓
Regulation of macromolecule biosynthetic process	✓	.	.	✓	✓	.	✓	.	✓	✓
Regulation of nucleobase, acid metabolic process	✓	.	.	✓	✓	.	✓	.	✓	✓
Pigment biosynthetic process	✓	.	✓	.	.	✓	✓	✓	✓	.
Regulation of biosynthetic process	✓	.	.	✓	✓	.	✓	.	✓	✓
Regulation of nitrogen compound metabolic process	✓	.	.	✓	✓	.	✓	.	✓	✓
Regulation of gene expression	✓	.	.	✓	✓	.	✓	.	✓	✓
Regulation of cellular metabolic process	✓	.	.	✓	✓	.	✓	.	✓	✓
Regulation of primary metabolic process	✓	.	.	✓	✓	.	✓	.	✓	✓
Regulation of macromolecule metabolic process	✓	.	.	✓	✓	.	✓	.	✓	✓
Regulation of metabolic process	✓	.	.	✓	✓	.	✓	.	✓	✓
Response to chitin	✓	✓	.	✓	.	.	✓	.	✓	✓
Regulation of cellular process	✓	.	.	✓	✓	.	✓	.	✓	✓
Response to osmotic stress	✓	.	.	✓	✓	.	✓	.	✓	.
Response to ethylene stimulus	✓	.	.	✓	✓	.	✓	.	✓	.
Regulation of transcription, DNA-dependent	✓	.	.	✓	✓	.	✓	.	✓	.
Regulation of RNA metabolic process	✓	.	.	✓	✓	.	✓	.	✓	.
Chlorophyll biosynthetic process	✓	.	✓	.	.	.	✓	✓	✓	.
Porphyrin biosynthetic process	✓	.	✓	.	.	.	✓	✓	✓	.
Tetrapyrrole biosynthetic process	✓	.	✓	.	.	.	✓	✓	✓	.
Regulation of biological process	✓	.	.	✓	.	.	✓	.	✓	✓
Red or far-red light signalling pathway	.	.	✓	.	✓	.	.	✓	✓	✓
Cellular response to radiation	.	.	✓	.	✓	.	.	✓	✓	✓

The rows contain different GO terms, and the columns represent 10 ecotypes. A ‘✓’ sign represents statistically significant (Hypergeometric test, Benjamini & Hochberg False Discovery Rate FDR correction, significance level 0.05) overrepresentation of that GO term in corresponding ecotype in that column. Due to space limitations, only some of the interesting GO terms overrepresented in multiple ecotypes were included in this table. The complete result obtained from the BiNGO analysis has been presented in the Additional file 3.

#### ***Cold response is coupled with light stimulus***

Along with the general cold response pathways or processes, there were several overrepresented categories related to ‘response to light’. Few genes in these categories were as follows- At1g29920 (*LHCB1*), At5g24470 (*PRR5*), At4g08920 (*OOP2*), At1g02340 (*RSF1*), At1g06040 (*STO*), At3g27690 (*LHCB2.4*), At3g54720 (*PT*), At2g42540 (*COR15A*), At2g26990 (*FUS12*), At5g24120 (*SIGE*), At2g46970 (*PIL1*), At4g18130 (*PHYE*), At5g67030 (*ZEP*), At5g45340 (*CYP707A3*), At1g02400 (*GA2OX6*), At2g46790 (*TL1*), At3g28860 (*PGP19*) At2g46340 (*SPA1*), At4g19230 (*CYP707A1*), At2g18790 (*PHYB*). Light and cold signals is known to integrate and cross talk for cold tolerance, via a CBF and ABA-independent pathway [54]. Franklin *et al.*[55] investigated the modulation of low R/FR signalling by ambient temperature and results showed that a low red to far-red ratio (R/FR) light signal increases CBF gene expression in *A. thaliana* in a manner dependent on the circadian clock. Red or far-red light signalling pathway is one of the significantly up-regulated GO categories in some of the ecotype [55]. Such signals stimulate expression of *CBF* genes through ambient temperature–dependent coupling of CBF transcription factors to downstream *COLD REGULATED* (*COR*) genes.

#### ***Chlorophyll biosynthetic process***

Another overrepresented GO category was chlorophyll biosynthetic process which included several genes like At1g78600 (*STH*3), At5g54190 (*PORA*), At3g59400 (*GUN4*), At3g56940 (*CRD1*), At4g34740 (*CIA1*), At1g78600 (*STH3*), At1g71030 (*MYBL2*), and At5g67030 (*ZEP*). Havaux *et al.*[56] reported that *A. thaliana* was able to survive in cold stress through light independent xanthophyll cycle by illustrating protective functions of carotenoid and flavonoid pigments against excess visible radiation at cold temperature [56]. Cold stress also induces synthesis of early light-induced proteins (ELIPs) [57]. Low temperature induces the accumulation of various antioxidants including carotenoids (except β-carotene), vitamin E (α- and γ-tocopherol) and non-photosynthetic pigments (anthocyanins and other flavonoids) [58]. Genes in the overrepresented category pigment biosynthetic process from our analysis support the previous reports.

#### ***Cold stress and circadian rhythms***

Circadian rhythm is one of the most prominent overrepresented categories in our dataset. It included many well-known genes belong to this category like At1g22770 (*GI*), At1g68050 (*FKF1*), At1g18330 (*RVE7*), At5g24470 (*PRR5*) [59], At5g17300 (*RVE1*), At2g46790 (*TL1*), and At2g46830 (*CCA1*). Previous studies reported a circadian clock regulated induction of *CBF* genes during low-temperature treatment in *A. thaliana* plants [60,61]. The circadian clock gates both gene expression and physiological responses to low R/FR during rapid shade avoidance [62,63]. Mikkelsen *et al.*[64] reported that cold- and clock-regulated gene expression are integrated through regulatory proteins that bind to Evening Element (EE) and Evening Element Like (EEL) elements supported by transcription factors acting at ABA response element (ABREL) sequences [64]. They established a role for circadian evening elements in cold-regulated gene expression in *A. thaliana*. Our current results are in good agreement with these previous reports.

#### ***Co-regulation of cold and biotic stress responsive genes***

Few categories in our gene set enrichment analysis (GSEA) were related to biotic stress response processes. Some of these categories were as follows -response to other organism, response to fungus, and response to bacterium, multi-organism process etc. Some of the up-regulated genes in these categories include At2g40140 (*ZFAR1*), At5g25110 (*CIPK25*), At5g25910 (*RLP52*), At1g20440 (*RD17/COR47*), At4g37150 (*MES9*), At3g50970 (*XERO2*), At2g42530 (*COR15B*), At2g44490 (*PEN2*), At5g64750 (*ABR1*), At1g51090, At4g12470, At4g36010 (*pathogenesis-related thaumatin family protein*), At3g51660 (*MIF family protein*), At1g20030 (*pathogenesis-related thaumatin family protein*), At3g50260 (*CEJ1*), At3g15210 (*RAP2.5*), At5g58600 (*PMR5*), At3g52400 (*SYP12*2), At3g06490 (*MYB108*), At1g19180 (*TIFY10A*), At4g23810 (*WRKY53*). Additional file 4 contains all the GO categories from each of ecotypes including the ecotype specific categories. One important observation was that biotic stress response related categories- response to other organism, response to fungus, response to bacterium, response nematode were overrepresented mainly in the southern ecotypes like Cvi, Kas1, Kyo2, Kond. Probable reason may be that plants from southern latitude often face such biotic invaders compare to their northern counterparts, and consequently have co-evolved with better and prompt defence response mechanisms against them. Based on genetic resources of *A. thaliana*, coupled with 39 years of field data, it has been reported that natural enemies drive geographic variation in plant defences [65].

Unlike cold tolerance, molecular mechanism of pathogen resistance obtained through cold treatment is not understood well. Plazek et al. reported that cold treatment on spring barley (*Hordeum vulgare.*), meadow fescue *(Festuca pratensis*) and oilseed winter rape (*Brassica napus var. oleifea*) induced resistance to their specific pathogens [66]. Zhu et al. identified a plant temperature sensitive component in disease resistance and provided a potential means to generate plants adapting to a broader temperature range [67]. Besides the available reports about enhanced disease resistance acquired through cold treatment, it is not yet known if these two traits are regulated by the same signal transduction pathway [68]. We have observed overrepresentation of GO categories like steroid hormone mediated signalling pathway, brassinosteroid mediated signalling pathway, jasmonic acid stimulus etc. Phytohormones are involved in induction of disease resistance upon pathogen infection. Plant hormones like salicylic acid (SA) ethylene (ET), jasmonic acid (JA) pathways are known to play important functions in the signal transduction during biotic stresses [69]. The occurrence of simultaneous biotic and abiotic stresses increases the complexity, as the responses to these are largely controlled by different hormone signalling pathways that may interact and inhibit one another [70]. Interaction of cold temperature and pathogen attack results potentially negative impact on plants [71]. Plants grow in heavy snowfall areas need to enhance disease resistance to survive from the attack of pathogens like snow molds [72]. Hence, as a nascent observation, the co-evolution of regulatory mechanism for co-occurring stress related genes and processes are highly possible. Further targeted screening of more ecotypes may find interesting results in this direction to explore interaction of biotic and abiotic stress on adaptive evolution of plant defence response.

### CBF regulon genes exhibits differential expression pattern in *Arabidopsis ecotypes* during cold treatment

**Figure 2:**
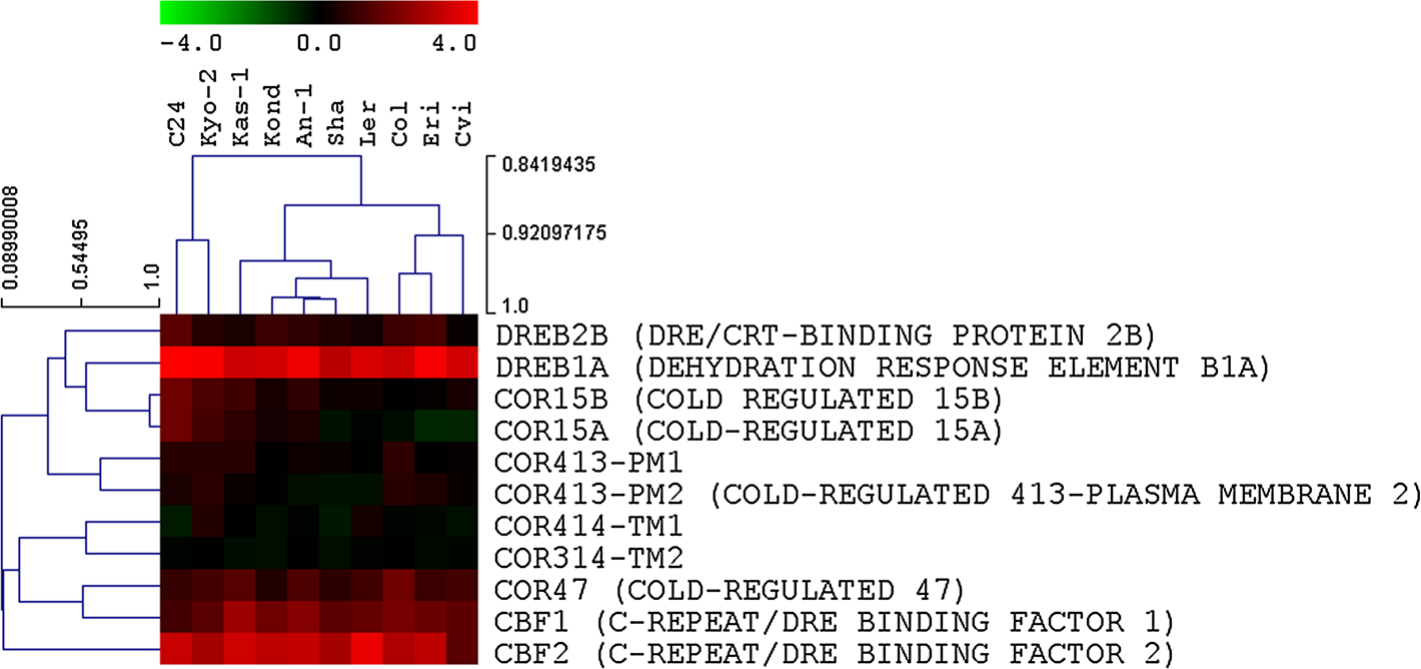
**Difference in gene expression among the CBF and COR genes.***CBF* genes as well as the selected *COR* genes were cold regulated in all accessions. But there were noticeable differences in the levels of expression among ten *A. thaliana* ecotypes.

The *A. thaliana* CBF cold response pathway has a major role in cold response. *CBF* genes appear to be present across plant species and are almost always present as a gene family. In *A. thaliana,* there are four characterized *CBF* genes: *CBF1, 2* and *3*, located on chromosome 4, are cold induced; except *CBF4* located on chromosome 5, which is reported to be involved in drought tolerance [73,74]. All the *CBF* genes as well as the selected *COR* genes were cold regulated in 10 ecotypes. But we observed different levels of expression of *CBF* and *COR* genes in the ten ecotypes. All the *CBF* genes were induced but *COR* genes had preferential expressions in different ecotypes (Figure 2). *DREBA1* expression consistently occurred in all the accessions. In a previously published study *CBF1* and *CBF2* were reported to have quite comparable expression levels in 9 ecotypes except low expression of both in Cvi [17]. Low expression of *CBF1, 2* in Cvi ecotype is clearly visible in our data (Figure 2). It was reported that expression of the CBF1, 2 and 3 genes was not correlated with cold tolerance level among ecotypes [59].

Variation in gene expression reflects the interplay between ‘robustness’ and ‘evolvability’; which is generally achieved by regulatory divergence. An organism has to preserve a consistent function under different conditions and at the same time, it needs to sustain the ability to evolve in order to adapt to new environments. The plasticity of gene expression may be achieved by selective accumulation of mutations in the promoter. As about 100 downstream genes and processes are regulated by the CBF and COR proteins, difference could be seen in the expression pattern of down-stream genes which was visible in the heat-map of 1000 genes and other ecotype specific differentially regulated genes (Figure 1). We chose to investigate the polymorphism present in the *CBF1, 2* and 3 genes and few *COR* genes using recently released data from *A. thaliana* 1001 genome project [75].

### Sequence polymorphisms seen in the *CBF* genes using data from *Arabidopsis thaliana* 1001 genome project

Sequence variation of *CBF* and *COR* genes could exert an effect at two different levels: either in the expression of the *CBF* genes themselves, via polymorphism in the respective promoter and/or in the expression of their downstream genes. It has been reported earlier that all three *CBF* genes were highly polymorphic, particularly in their promoters, with *CBF1* the most and *CBF2* the least polymorphic gene [17,76]. Hence, we have downloaded the sequence data (including 1kbp upstream of the coding region) available from the 1001 genome database and calculated Tajima’s D statistic to evaluate the allele frequency spectrum and quantify the excess of rare alleles. We observed significant number of non-synonymous amino acid changes in the coding region of the *CBF* genes (Additional file 5). The three *CBF*s have shown significantly negative Tajima’s D values (*CBF1* = −1.291, *CBF2* = −2.223, *DREBA1* = −2.165). More negative and significantly lower values of Tajima’s D indicate an excess of rare and recent, alleles [45]. But, it is known from earlier studies that the average distribution of Tajima’s D in the *A. thaliana* genome is known to be biased towards negative values [77-79]. We could not conclude any direct correlation between sequence polymorphism on gene expression pattern of the *CBF* and *COR* genes. The non-existence of a clear correlation between *CBF* and *COR* gene expression with sequence polymorphism in 10 ecotypes might have several reasons. There are other CBF independent pathways and their complex interactions between different components contribute to cold tolerance [12]. So, how these complex interactions of other pathways affect *CBF* and *COR* gene expression would be difficult to predict. Again, *COR* genes are often up-regulated much later, and this is also true for protein expression. Gene expression without protein synthesis can not generate downstream product. Apart from genotype variation, the length of cold exposure and treatment temperature also affect the gene expression level that leads to freezing tolerance [80]. While studying natural variation of transcriptional auxin response networks in *A. thaliana*, Delker et al. reported that differentially regulated signalling networks had a greater role to play than sequence polymorphism [81]. Considering such facts, we wanted to explore the pattern of regulatory divergence of cold stress response network among these ten *A. thaliana* ecotypes.

### Reverse engineering transcriptional regulatory network during cold stress response in *A. thaliana*

**Figure 3:**
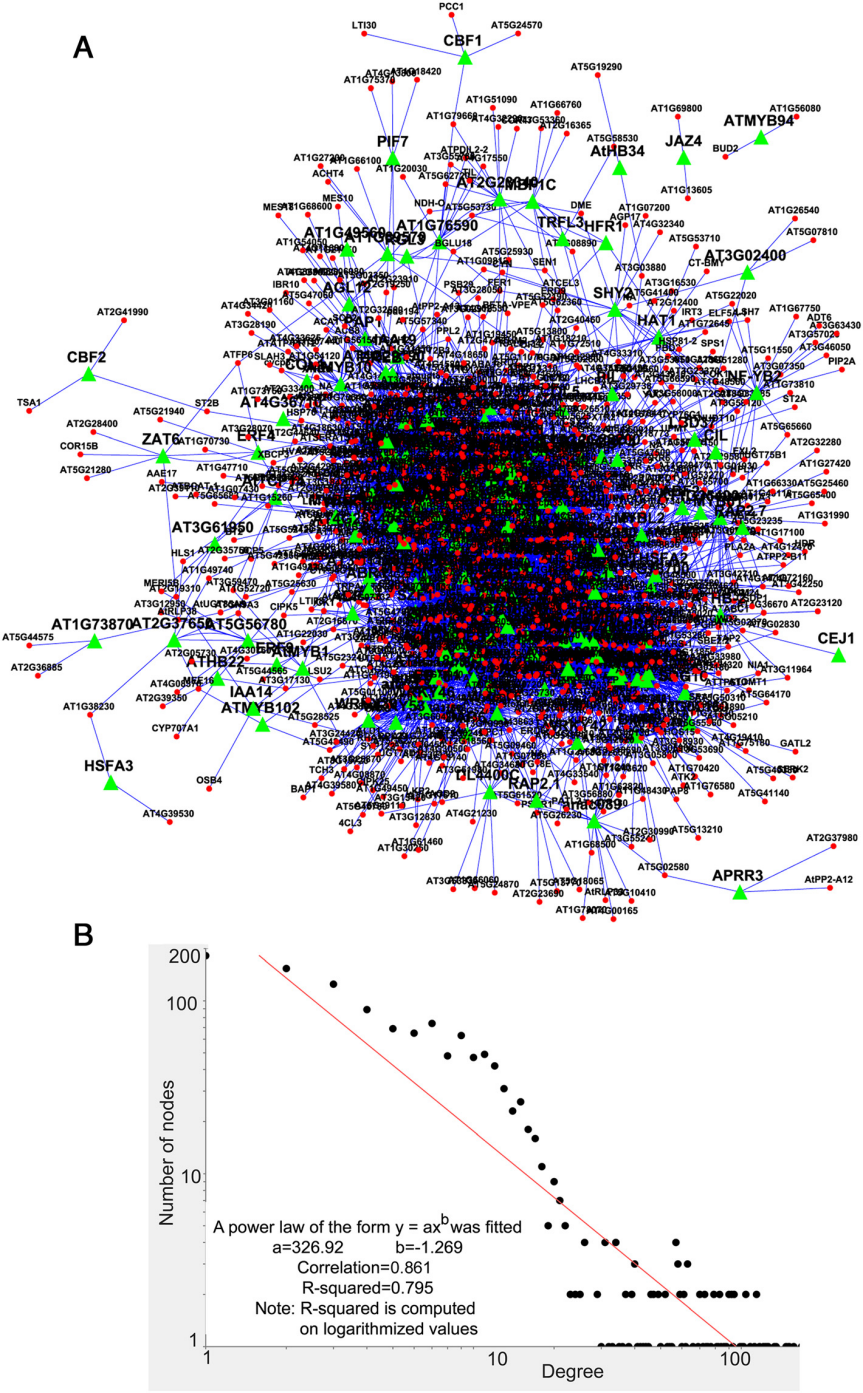
**Transcriptional regulatory network constructed using cold stress microarray data from 10 *A. thaliana *ecotypes. (A)** Topological overview of the constructed network**.** The network contains 1,275 genes (nodes) and 7,720 connections. Transcription factors are marked as green triangles and target genes are marked as red circles. Predicted regulatory interactions are shown as arrow (→) for activation (6,731) and down-horizontal bar (┤) as repression (989). Network was visualized in Cytoscape software using ‘Force-Directed Layout’. **(B)** Scale-free behaviour of the predicted network. This plot shows the power-law degree distribution of the network *P (k)* at correlation thresholds (r ≥ 0.8). Here *k* indicates connectivity, and *P (k)* indicates the connectivity distribution of the genes (nodes) in the network. This satisfies the existing notion about scale free behaviour of biological networks. Few TFs in the network are highly connected (hubs) than others.

Due to the lack of experimentally validated transcriptional regulatory information in *A. thaliana*, we have decided to reverse engineer an in-silico transcriptional regulatory network model during cellular responses to cold stress in *A. thaliana* using our gene expression data. For this purpose, we have selected top 1,509 differentially cold regulated transcripts from the union of the entire cold regulated transcripts list, considering a criterion that a transcript had to be significantly regulated at least in 2 of the 10 ecotypes. The resulting list contained 178 TFs and 1,331 target genes (TGs). By using NCA method (explained in materials and methods), we have constructed the network at correlation-coefficient threshold ≥ 0.8. Activation and repression interactions were calculated using the positive and negative correlations. The resulting network contained 1,275 nodes and 7,720 connections; out of them 6,731 connections were activations (positive) and 989 were repressions (negative) (Additional file 6 and Figure 3A). Some of the highly connected positive regulators (TFs) were *ATTLP7, POSF21, AS1, RTV1, APRR9, BT1, ANAC102, ANAC035, GLK2, ZFN1, WRKY11, HAC5, MYB73, DA1, LBD41, SR1, WRKY70*. Further details of the constructed network including calculated Pearson’s correlation coefficient and *p-values* have been given in Additional file 6. In the network visualization, transcription factors were marked as green triangles and target genes were marked as red circles. General network topology based analysis has revealed that the network exhibited power-law degree distribution [82] (Figure 3B). We have also calculated several other graph-theoretic parameters as described by Barabasi et al. [83]. Some of the parameters were as follows- clustering coefficient = 0.3, connected components =3, network diameter = 11, characteristic path length = 3.67, average number of neighbours = 11.385. The constructed network satisfies the existing notion about scale free behaviour of biological networks [84]. Few of the TFs in the network are highly connected (hubs) than others. The generated network (.cys file) has been provided as Additional file 7. Interested reader can locally open the file using Cytoscape software [85] and do more interactive exploration. The presented view of the annotated network in this manuscript has been simplified manually.

### Correlation between activity and expression values of TFs

Simple correlation between the expression profile of a transcription factor and its targets is not obvious, and simple clustering based methods have not been very successful in deciphering them [86]. The key assumption during prediction of interactions between TFs and their target genes using gene expression data is that high dimensional mRNA expression profiles contain hidden regulatory signals, which can be decomposed to low-dimensional regulatory signals driven through an interacting network [87]. The lower dimensional regulatory signals can be represented as a bipartite networked system of the transcription factors and the target genes, where the gene expression levels are transformed into weighted functions of the intracellular states corresponding to the activity of the transcription factors [31,33]. Several such methodologies have been used in plant systems to infer regulatory relationships at various occasions [23,33,88-91].

The NCA algorithm requires two inputs to calculate the hidden regulatory activity profiles: a series of gene expression profiles and a pre-defined regulatory network. The *A. thaliana* transcription factor list were collected from the Database of Arabidopsis Transcription Factors (DATF) [25], The Arabidopsis Gene Regulatory Information Server (AGRIS) [26], and the Plant Transcription Factor Database (PlantTFDB) [92]. List of 59 cold regulated transcription factors were collected from Gene Ontology database under the annotation category ‘response to cold’ [43]. The constructed network was able to capture 30 (~50%) of these already reported cold regulated TFs.

### Transcription factor activity under cold stress in different *Arabidopsis* ecotypes

**Figure 4:**
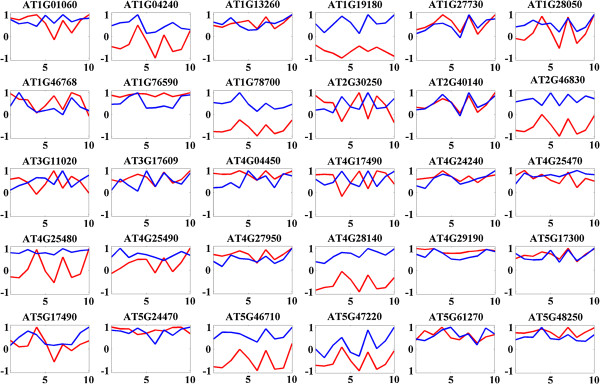
**Differential activity profiles of 30 known cold regulated transcription factor in ten ecotypes of *A. thaliana *predicted using NCA algorithm.** Rows represent the TFs and columns different eco-types response to cold treatment. Transcription factor activities were shown in blue their expression values were represented as red colour. Here values are scaled for direct comparison purposes. X-axis represents the different eco-types (1 = Cvi, 2 = Kas1, 3 = Kyo.2, 4 = Col, 5 = Kond, 6 = Sha, 7 = C24, 8 = Ler, 9 = An.1, 10 = Eri).

We have compared the predicted activities of the 30 previously reported cold responsive transcription factors with their corresponding gene expression values in the ten *A. thaliana* ecotypes. About 57% of transcription factors showed moderate correlation (Pearson correlation coefficient, |r| > 0.5) between their activities and expressions (Figure 4). Thirteen TFs ((*ZFAR1* (At2G40140), At1G28050, *ERF2* (At5G47220), *ZAT10* (At1G27730), At5G46710, *CRF4* (At4G27950), At1G78700, At5G17300, At4G28140, At5G48250, At4G29190, *RAP2* (At1G46768), and *WRKY7* (At4G24240)) exhibited positive correlations (r > 0.5). For instance CZF-1 (At2G40140) had correlation r = 0.9206 which indicated that there might be chance of auto regulation. This assumption was supported by literature [93] and information available from the Arabidopsis Gene Regulatory Information Server (AGRIS). Four transcription factors ((*WRKY25* (At2G30250), *ERF6* (At4G17490), *DREB2B* (At3G11020) and *TIFY10A* (At1G19180)) showed strong negative correlation (r < −0.5) and the remaining TFs displayed low or no correlation at all (|r| < 0.5). Three of these predictions have been confirmed from AGRIS database.

The predicted activity profiles of thirty cold regulated TFs have clearly shown the ecotype specific variations in the ten *A. thaliana* ecotypes (Figure 4). For example, transcription factor At5G17300 (*RVE1*) was highly active in Eri, C24 and Col ecotypes compared to the others. Most of the transcription factors were active in more than two ecotypes (Table 4). We have also identified ecotype specific transcription factors (highly active in single ecotype). Transcription factors At1G04240 (*SHY2*), At2G46830 (*CCA1*) and At3G11020 (*DREB2B*) were active in Sha ecotype and transcription factor At4G25490 (*DREB1B/CBF1*) was more active in Eri ecotype. Spatio-temporal regulatory dynamics of *SHY2*[94] and *CCA1*[95,96] have been reported earlier. The transcription factor At5G17490 (*RGL3*) [97] was active in Col-0 ecotype. We found a group of transcription factors, which were highly active in a particular set of ecotypes. For example, transcription factors At1G27730 (*ZAT10*), At1G28050, At3G17609 (*HYH*), AtAt4G27950 (*CRF4*), At5G17300 and At5G48250 were active in Eri, C24 and Col-0 ecotypes and transcription factors At1G9180 and At4G25480 were highly responsive in Eri and Col-0 ecotypes during cold treatment. All the *A. thaliana* ecotypes had at least 7 (out of 30 core cold responsive TFs) active TFs, except the Kond ecotype. This ecotype had only two significantly active TFs (At1G76590 and At4G04450).

**Table 4:** Ecotype specific transcriptional activity profiles of the 30 cold responsive TFs

**TF ID**	**Alias**	**Ecotypes**
At1G01060	LHY1	Eri, Col-0, Cvi, Kyo-2
At1G04240	SHY2	Sha
At1G13260	RAV1	Eri, C24
At1G19180	TIFY10A	Eri, Col-0
At1G27730	ZAT10	Eri, C24, Col-0
At1G28050	At1G28050	Eri, C24, Col-0
At1G46768	RAP2.1	An-1, Sha, L*er*, Cvi
At1G76590	At1G76590	Eri, Kond, C24, An-1, Col-0, Sha, L*er*, Cvi, Kyo-2, Kas-1
At1G78700	At1G78700	Sha, L*er*, Cvi, Kas-1
At2G30250	WRKY25	Sha, L*er*, Cvi
At2G40140	ZFAR1/CZF1	Eri, C24
At2G46830	CCA1	Sha
At3G11020	DREB2B	Sha
At3G17609	HYH	Eri, C24, Col-0
At4G04450	WRKY42	Eri, Kond, C24, An-1, Col-0, Cvi, Kyo-2, Kas-1
At4G17490	ERF-6-6	An-1, Sha, L*er*, Cvi, Kyo-2, Kas-1
At4G24240	WRKY7	Eri, Col-0
At4G25470	CBF2	C24, Col-o, Kas-1
At4G25480	DREB1A	Eri, Col-0
At4G25490	DREB1B/CBF1	Eri
At4G27950	CRF4	Eri, C24, Col-0
At4G28140	At4G28140	An-1, Sha, L*er*, Cvi, Kas-1
At4G29190	At4G29190	Eri, An-1, Cvi, Kyo-, Kas-1
At5G17300	At5G17300	Eri, C24, Col-0
At5G17490	RGL3	Col-0
At5G24470	PRR5	An-1, L*er*, Cvi, Kyo-2, Kas-1
At5G46710	At5G46710	An-1, Sha, L*er*, Cvi, Kas-1
At5G47220	ERF2	Sha, L*er*, Kas-1
At5G48250	At5G48250	Eri, C24, Col-0
At5G61270	PIF7	Col-0, Kas-1

This table presents, which of the 30 previously reported cold responsive TFs are active among ten ecotypes during our experiments based on their predicted activity profiles using NCA algorithm.

## Conclusions

Here we undertook an experiment to analyze the natural variation in genome-scale cold stress response regulatory networks in ten *A. thaliana* ecotypes at a single time point (3 hours) gene expression measurement. The analysis indicated that the 10 *A. thaliana* ecotypes had different transcriptome level signatures in response to non-freezing cold stress. Col-0 being known as cold tolerant ecotype had significantly less number of differentially regulated transcripts while Cvi as the most southern most ecotype had the highest number of differentially regulated transcripts. Among the differentially cold regulated transcripts, 75% showed ecotype specific expression pattern. There were 315 transposable elements (TEs) in the ecotype specific differentially regulated gene list. These TEs may play an important role in plant genome evolution while adapting to local climatic temperatures. *CBF* genes were cold induced in all the ecotypes, irrespective of their geographic origin. But their levels of expressions varied among different ecotypes. Expression pattern of the *COR* genes were not consistent in all ecotypes. Sequence data available from the 1001-genome project indicated that the mutations in their sequences might contribute to the dramatic difference in the expression pattern. Significant numbers of non-synonymous amino acid changes were observed in the coding region of all the *CBF* genes. All of the *CBF*s had shown significantly negative Tajima’s D values, indicating an excess of rare and recent, alleles. Gene ontology analysis had shown that apart from common cold stress regulated processes; several other biological processes were differentially regulated in the 10 ecotypes. Some of the important GO categories were - pigment biosynthesis, circadian rhythm, response to light, response to water deprivation, response to ABA. By looking at the differentially regulated genes related to pathogen responses induced by cold stress, a primary assumption was made that the co-evolution of severely affecting co-occurring stress related genes and processes was highly possible. We have constructed an *in silico* transcriptional regulatory network model during cellular responses to non-freezing cold stress in *A. thaliana,* using gene expression data from 10 ecotypes. The network contained 1,275 nodes and 7,720 connections, which included 178 TFs and 1,331 target genes. Apart from retaining several previously known interactions (cross validated using AGRIS), many novel regulatory interactions during the cold stress response in *A. thaliana* were suggested. Differential regulatory activities were observed among the cold regulated TFs, which might contribute towards cold adaptation of the ecotypes. In addition, since the approach is general, it could in principle be used to study networks regulating biological process in any biological systems. As far as cold stress is concerned, it could be implemented for identification of useful molecular markers or relevant forward genetics experiments to develop cold tolerant crop varieties.

## Competing interests

The authors declare that they have no competing interests.

## Authors’ contributions

JM, AMB and HBN conceived the multistress project. PB developed the concept of the study, performed bioinformatics analyses and drafted the manuscript. NDJ performed the NCA analysis. SR and HBN were responsible for the microarray experiments and data pre-processing. JM was leading the ERA-NET PG Multi-Stress project and contributed towards improvement of the manuscript. AMB coordinated the overall development of the manuscript. All authors have read and approved the manuscript.

## Supplementary Material

Additional file 1 **List of all transcripts from 10 ecotypes with annotations, p-values and fold change values during cold treatment.** United list of 6061 differentially cold regulated transcripts and 498 TFs are in separate sheets of the same excel file. Click here for file

Additional file 2 List of statistically significant ecotype specific gene expressions during cold treatment from 10 ecotypes are presented in 10 different sheets. Click here for file

Additional file 3 **Results of Gene Set Enrichment Analysis using BinGO software.** The individual results for each of the 10 ecotypes have been put in a single file. Each analysis contains the detailed statistical test, significance score for each GO-term and corresponding gene IDs in that category. Click here for file

Additional file 4 **GO category contingency table from the significantly regulated gene-list, generated using BiNGO software.** The rows contain different GO terms, and the columns represent 10 ecotypes. A ‘✓’ sign represents statistically significant (Hypergeometric test, Benjamini & Hochberg False Discovery Rate FDR correction, significance level 0.05) overrepresentation of that GO term in corresponding ecotype in that column. The column with the header ‘occurrence count’ represents in how many ecotypes the respective term is significantly over represented. Click here for file

Additional file 5 Analysis of sequence polymorphism in CBF and COR genes. Click here for file

Additional file 6 **TF-TG regulatory bipartite connections predicted using NCA algorithm based on their activity profiles, using Pearson correlation coefficient threshold (PCC) ≥0.80.** The second sheet (TF_ACT_REP) includes predicted pattern of regulation (activation or repression) in the network. Click here for file

Additional file 7 **Predicted TF-TG regulatory networks as a .cys file, which can be open locally by a reader for interactive exploration.** For visualizing the network locally, download cytoscape software from http://www.cytoscape.org/ and load the .cys files on the software. The presented view of the annotated network in this manuscript has been simplified manually for representation purpose. Click here for file
