# Supplementary material for: Genome-scale cold stress response regulatory networks in ten Arabidopsis thaliana ecotypes
Source: BMC Genomics. 2013 Oct 22;14:722. doi: 10.1186/1471-2164-14-722 (PMC3829657; doi:10.1186/1471-2164-14-722)
Supplement: Additional file 5 — Analysis of sequence polymorphism in CBF and COR genes. [file 1471-2164-14-722-S5.doc]

**(S5)**

**Results from Tajima's Neutrality Test [1]**

| **Genes** | m | S | ps | T | p | D |
| --- | --- | --- | --- | --- | --- | --- |
| **CBF1** | 501 | 680 | .0687560 | .0101220 | .005514 | -1.291374 |
| **CBF2** | 496 | 590 | .0595360 | .0087770 | .001834 | -2.222324 |
| **CBF3/DREBA1** | 476 | 700 | .0704930 | .0104570 | .002521 | -2.164592 |

*****Abbreviations: m = number of sequences, S = Number of segregating sites, ps = S/m, T = ps/a1, p = nucleotide diversity, and D is the Tajima test statistic (see chapter 12 in ref. [3] for details).

The analysis involved nucleotide sequences downloaded from Salk Arabidopsis thaliana 1001 Genomes (<http://signal.salk.edu/atg1001/accessions.php>). Initially sequences from all available ecotypes (724) were downloaded, but incomplete sequences were discarded before the analysis. Apart from the coding regions we have considered 1000 bp upstream sequences for alignment. We have considered Codon positions included were 1st+2nd+3rd+Noncoding. All positions containing gaps and missing data were eliminated. Evolutionary analyses were conducted in MEGA5 [2,3]. A negative Tajima's D signifies an excess of low frequency polymorphisms relative to expectation, indicating population size expansion (e.g., after a bottleneck or a selective sweep) and/or purifying selection. A very rough rule of thumb to significance is that values greater than +2 or less than -2 are likely to be significant. This rule is based on an appeal to asymptotic properties of some statistics, and thus +/- 2 does not actually represent a critical value for a significance test.

**REFERENCES:**

1. Tajima F. (1989). Statistical methods to test for nucleotide mutation hypothesis by DNA polymorphism. Genetics 123:585-595.

2. Tamura K., Peterson D., Peterson N., Stecher G., Nei M., and Kumar S. (2011). MEGA5: Molecular Evolutionary Genetics Analysis using Maximum Likelihood, Evolutionary Distance, and Maximum Parsimony Methods. Molecular Biology and Evolution (In Press).

3. Nei M. and Kumar S. (2000). Molecular Evolution and Phylogenetics. Oxford University Press, New York.

**Sequence Polymorphisms seen in the CBF genes (coding regions)**

We observed significant number of non-synonymous amino acid changes in the coding region of the CBF genes.

**Nucleotide**

A SNPs -- Red line
C SNPs -- Blue line
G SNPs -- Green line
T SNPs -- Yellow line
1 bp deletions -- Black line
Unsequenced regions -- .. (dot) or grey area

**Amino Acide**

Synonymous amino acid -- Green line
Non-synonymous amino acid -- Red line

All standard amino acide codes are used. In addition, a Z - indicates an unknown amino acid, possibly a deletion and an X - indicates an amino acid has become a stop codon.

1) DREB2B (DRE/CRT-BINDING PROTEIN 2B)


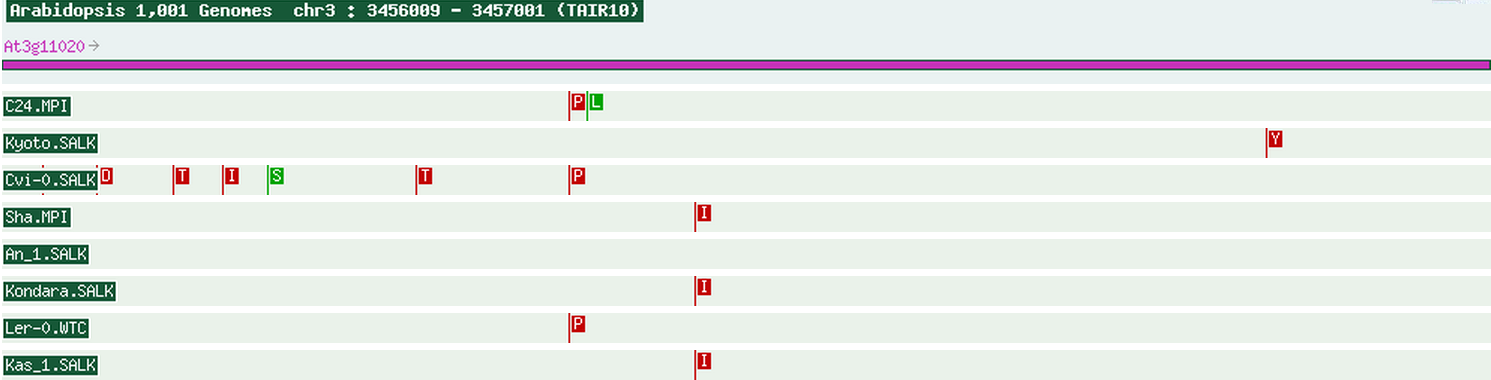
Nucleotide


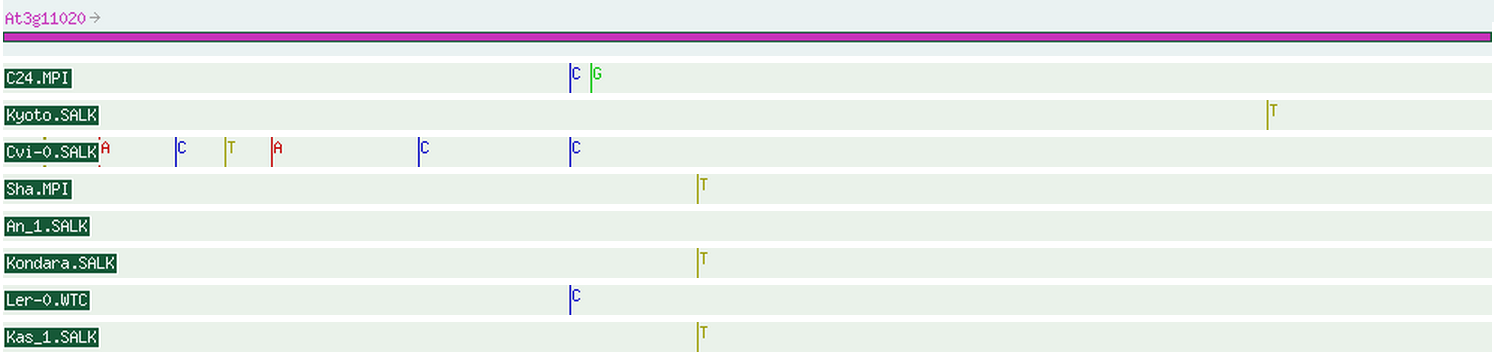
Amino-acid

2) CBF2 (C-REPEAT/DRE BINDING FACTOR 2)


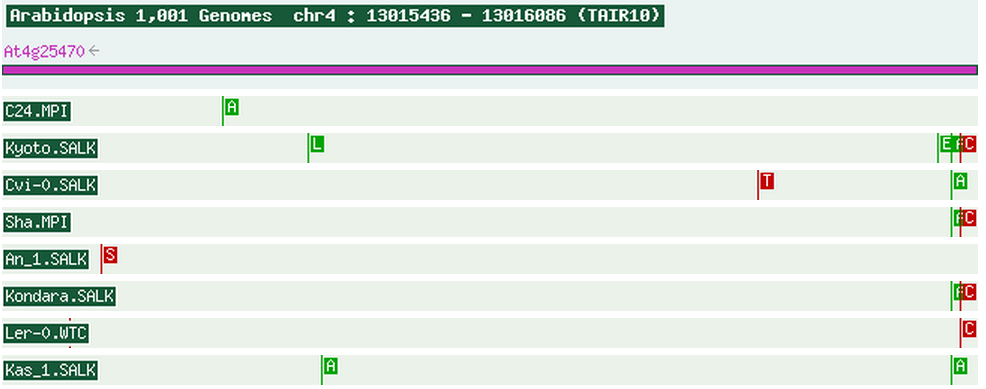
Nucleotide


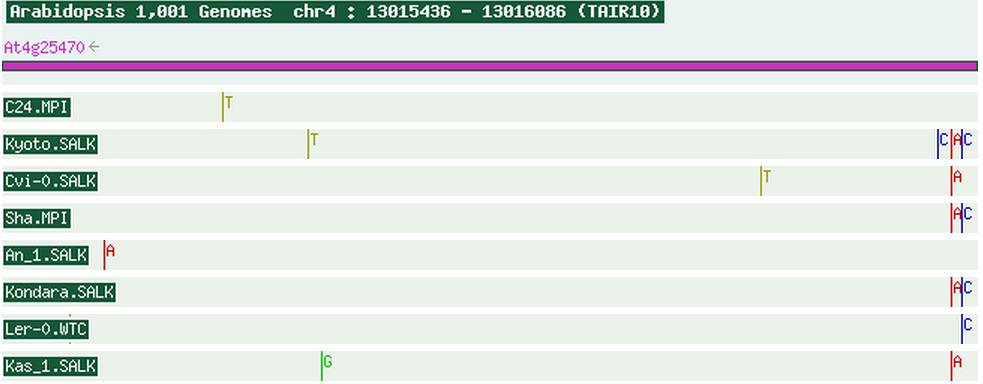
Amino-acid

3) DREB1A (DEHYDRATION RESPONSE ELEMENT B1A)

Nucleotide


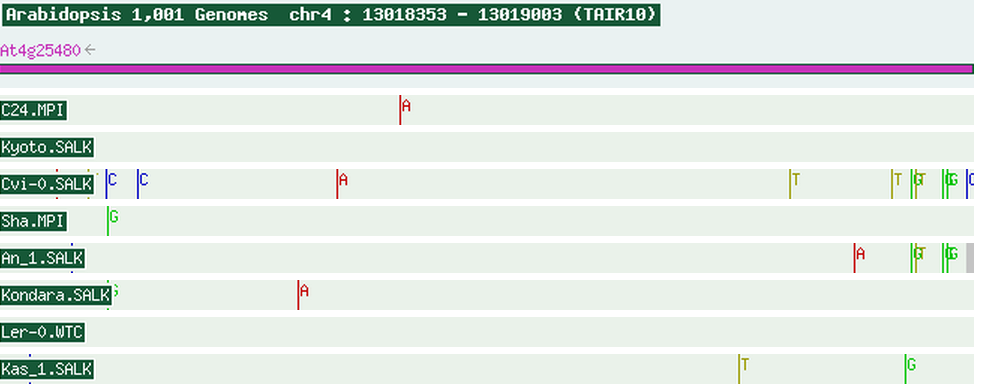


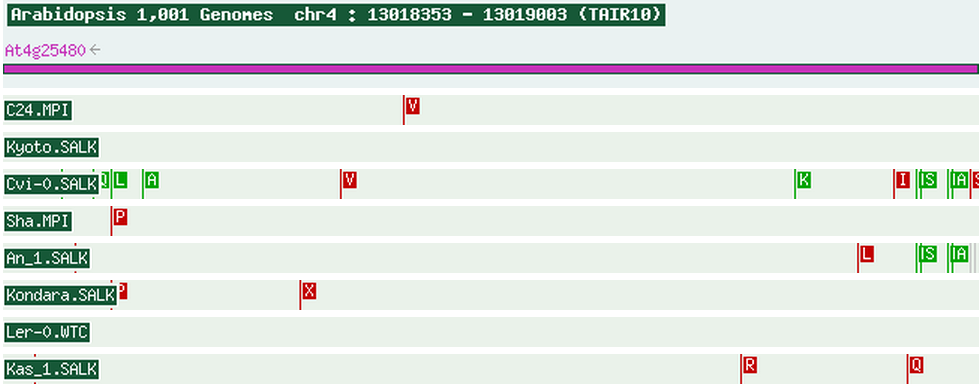
Amino-acid

4) CBF1 (C-REPEAT/DRE BINDING FACTOR 1)


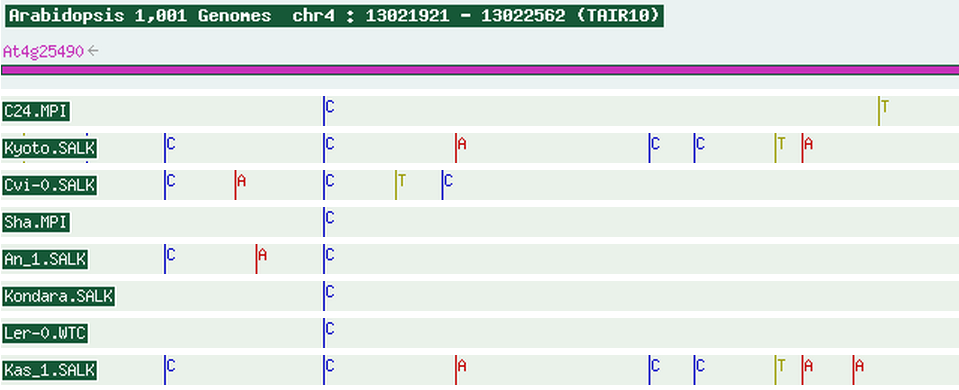
Nucleotide


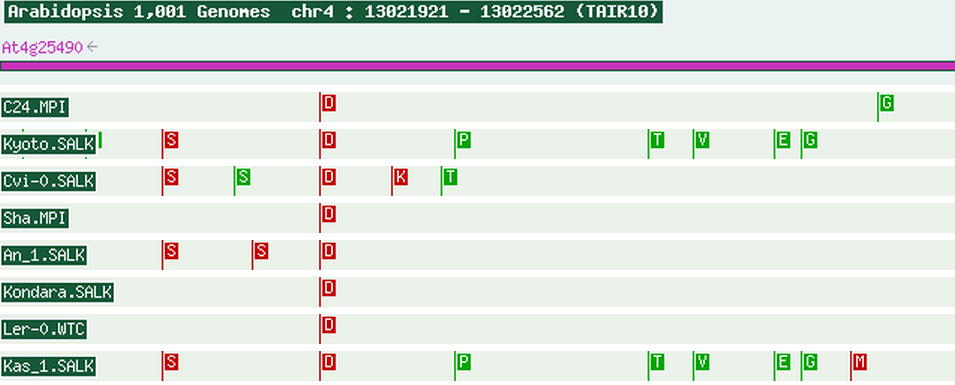
Amino-acid
